# Children from Baffin Island have a disproportionate burden of tuberculosis in Canada: data from the Children's Hospital of Eastern Ontario (1998-2008)

**DOI:** 10.1186/1471-2431-10-102

**Published:** 2010-12-30

**Authors:** Michael Clark, Charles Hui

**Affiliations:** 1 Department of Pediatrics, Children's Hospital of Eastern Ontario, Ottawa, Canada

## Abstract

**Background:**

The Children's Hospital of Eastern Ontario (CHEO) provides services to children in Baffin Island, through the Baffin Island Pediatric Health Initiative. Tuberculosis (TB) remains a major public health problem in that region. The objective of our study was to describe the origin and clinical characteristics of patients with TB disease at CHEO, since the inception of the Baffin Island Pediatric Health Initiative.

**Methods:**

All charts with a discharge diagnosis of TB disease during the first 10 years of the Baffin Island program were reviewed. Patients meeting a pre-determined case definition were included in analyses. A standard medical record abstraction form was used for patient data collection.

**Results:**

Twenty patients met our case definition. Seven (35%) were Canadian-born children from Baffin Island. Seven resided in Ontario, 4 in Quebec, and 2 were visiting from other countries. All 7 children residing in Ontario were born in African countries. Endothoracic disease occurred in 16 patients (80%), including 9 with primary pulmonary TB, and 3 with sputum smear positive "adult-type" disease. Extrathoracic disease was present in 6 children (30%), including 3 with CNS disease. Three children had disease in 2 separate sites.

**Conclusions:**

While Baffin Island makes up 1% of the hospital catchment population, they contributed 35% of TB patients, and the only TB death. While TB in foreign-born children is due in part to epidemics abroad, the problem in Baffin Island is a reflection of disease burden and transmission within Canada.

## Background

The Children's Hospital of Eastern Ontario (CHEO) provides services to children living in parts of eastern Ontario, western Quebec, and Baffin Island. The latter jurisdiction lies within Nunavut, a territory of northern Canada since 1999. The majority of people living in Baffin Island are of Inuit origin. Nunavut - "our land" in the Inuktitut language - was formerly the eastern most part of the Northwest Territories. The government of Nunavut is responsible for the provision of primary health care services in Baffin Island, maintained through the Nunavut Department of Health and Social Services. The role of CHEO is one of clinical support, through the Baffin Island Pediatric Health Initiative. This program has been in place since April, 1998. Services are provided directly by pediatric residents from CHEO, who rotate through Baffin Island as part of their training program. Direct services are also provided by visiting subspecialists, such as in pediatric cardiology, and through telephone advice to local physicians. Patients in need of critical care, surgery, specialized diagnostics, or tertiary medical care, are transported to CHEO for admission or outpatient care. In these situations, temporary housing is provided to the patient and/or family. Finally, ongoing education is provided by staff at CHEO to health care workers throughout Baffin Island via teleheath services.

CHEO provides services to an estimated population of 600, 000 children. In 2006, the overall population of Baffin Island was 15, 765. The population aged 0-17 years was 6065 [1], comprising approximately 1% of the estimated CHEO catchment population.

We conducted a retrospective chart review of TB inpatients at CHEO over a 10-year period. The objectives of the study were: 1) to describe the relative contribution of different geographical areas - both in terms of residence and original birthplace - to our inpatient population since the start of the Baffin Island program; and 2) to describe the clinical manifestations, diagnostic methods, and clinical course of TB inpatients at CHEO.

## Methods

Ethics approval for the chart review was obtained from the CHEO Research Ethics Board. Charts of all patients with an admission and/or discharge diagnosis of TB from April, 1998 to March, 2008 were reviewed. This included all cases with ICD-9 010-018 until March, 2002, and all cases with ICD-10 A15-A19 from April, 2002 onwards. Prior to reviewing the charts, a case definition was developed (Table 1) following review and consideration of the Canadian case definition [2] and a recent review [3]. If patients did not meet the criteria in Table 1, they were not included in subsequent analyses. A total of 28 charts were reviewed, of which 20 met the case definition for TB.

**Table 1:** Criteria for confirmed pediatric TB case in chart review

A) Culture isolation of *Mycobacterium tuberculosis *from patient specimen OR B) Radiological findings consistent with TB AND 2 or more of: 1) a positive TST; 2) confirmed contact with an infectious case; 3) a specimen positive on microscopy or molecular testing; 4) CSF findings consistent with TB^a^ OR C) All of: 1) Radiological findings consistent with TB; 2) no diagnosis more likely than TB; and 3) clinical improvement on antitubercular therapy AND one or more of: 1) a positive TST; 2) confirmed contact with an infectious case; 3) a specimen positive on microscopy or molecular testing; 4) CSF findings consistent with TB^a^

^a^3 or more of: 50-500 leukocytes; a lymphocyte predominance; glucose <50% of serum level; elevated protein

TST: tuberculin skin test; CSF: cerebrospinal fluid

Specimens for *Mycobacterium tuberculosis *culture are sent from CHEO to the Ottawa Regional Public Health Laboratory (ORPHL), where culturing is done using the BACTEC MGIT system. Smear microscopy of specimens is done at CHEO and at the ORPHL. Acid-fast staining is done with Kinyoun stain at CHEO. At the ORPHL, concentrated staining is done by the fluorochrome auramine method, and confirmed by Kinyoun stain. Specimens are sent to the Toronto Regional Public Health Laboratory for molecular testing, which consists of the AMTD^® ^nucleic acid amplication test. Microbiological testing was done elsewhere in a number of patients, in which a work-up had been initiated prior to transfer to CHEO. Patients were only considered positive for culture, smear, or molecular testing if a confirmatory report was available in the chart or from the laboratory.

Chest x-ray (CXR) and other relevant imaging reports were reviewed for findings consistent with TB [4-7]. If the interpretation in a given report was unclear, the original image was reviewed with a paediatric radiologist. A tuberculin skin test (TST) was considered positive if the result met criteria from the Canadian Tuberculosis Standards [8]. The definition for "contact" used in the study was derived from the same document. To meet our criteria for contact in Table 1, the infectious source case had to be diagnosed with TB of the respiratory system through isolation of *M. tuberculosis *from sputum or other respiratory specimen. Contact with such cases was verified with the public health nurse at Ottawa Public Health, City of Ottawa, or the Health Protection Unit, Department of Health and Social Services, Nunavut.

A standard medical record abstraction form was developed for data collection [9]. The information collected included demographic data, clinical manifestations at presentation, bacille Calmette-Guérin (BCG) vaccination history, human immunodeficiency virus (HIV) status, results of diagnostic investigations, hemoglobin and mean corpuscular volume (MCV) values, complications, and surgical interventions. The form was developed after a review of the clinical manifestations, complications, diagnosis and management of TB [10]. Following this review, lists were created for all qualitative variables, including symptoms, physical findings, CXR findings, specimens sent for microbiology, acute and chronic complications, and surgical interventions.

BCG vaccination status can be assessed on history, physical examination (presence/absence of a BCG scar), or by review of immunization records. Among those children considered recipients, we recorded the criteria used from most to least reliable (i.e. 1) availability of records, 2) presence of a scar, or 3) verbal history). If a family denied BCG vaccination on history and neither of the other 2 criteria were fulfilled, the child was classified as a non-recipient. All others were classified as having unknown BCG status. Hemoglobin and MCV values were compared to age-specific normal ranges provided by the CHEO hematology laboratory, and were classified as normal, high or low. Patients with low hemoglobin and a normal MCV were classified as having normocytic anemia, while those with low hemoglobin and a low MCV were classified as having microcytic anemia. Mention of an HIV work-up, or at least consideration of co-infection, was present in many charts in the absence of further information or testing results. Testing is done at the ORPHL, and results are forwarded to the CHEO virology laboratory. Records at the CHEO virology laboratory were reviewed for all patients included in the study, to verify if testing was done and the results of testing.

## Results

Twenty children met our criteria for TB disease during the study period. These cases are summarized in Table 2. Details of the 8 cases excluded from the study are provided in Table 3. Among disease cases, both genders contributed equally. Eleven (55%) of cases were 10 years or older. Six (30%) were in the 0-4 year age range, and 5 of these children were aged one year at diagnosis. Four of the 6 children aged 0-4 years (67%) were from Baffin Island. The cases in Table 2 are presented in chronologic order. Cases 1-10 were admitted during the first five-year period of the study, while cases 11-20 were admitted during the second five-year period.

**Table 2:** Origin and diagnostic results of TB inpatients at CHEO (1998-2008)

Case/age (y)/sex		Residence	Birth country	PC	HIV status	TST (mm)	CXR	Cx	TB disease site(s)
1	17 m	Ottawa	Somalia	-	U	n/a	-	+	Spine
2	12 f	Ottawa	Somalia	-	-	26	+	+	Adult-type
3	14 f	Ottawa	Somalia	-	U	27	+	+	Abdominal
4	12 f	Baffin	Canada	+	U	27	+	+	Adult-type
5	1 f	Quebec	Canada	+	U	22	+	+	Primary pulmonary
6	14 m	UK	UK	-	U	n/a	+	+	Pleural
7	6 f	Ottawa	Somalia	-	U	16	+	+	Primary pulmonary
8	11 m	Quebec	Canada	-	-	19	+	-	Primary pulmonary
9	6 f	Ottawa	Rwanda	-	+	n/a	-	+	CNS and abdominal
10	8 f	Quebec	Canada	+	U	20	+	-	Primary pulmonary
11	15 f	Ottawa	Zambia	-	U	n/a	+	+	Pleural
12	13 m	Baffin	Canada	+	U	n/a	+	+	CNS and miliary
13	17 f	Ottawa	Somalia	-	U	n/a	+	+	Superficial LN and primary pulmonary
14	1 m	Baffin	Canada	+	-	25	+	-	Primary pulmonary
15	1 m	Baffin	Canada	+	U	22	+	+	Primary pulmonary
16	1 m	Baffin	Canada	-	U	40	+	+	Primary pulmonary
17	4 m	Baffin	Canada	+	U	n/a	-	-	CNS
18	1 m	Quebec	Canada	-	U	14	+	+	Primary pulmonary
19	14 f	Baffin	Canada	+	U	22	+	-	Pleural
20	17 m	Haiti	US	-	-	20	+	+	Adult-type

PC: proven contact; TST: tuberculin skin test; CXR: chest x-ray; Cx: culture; m: male; f: female; U: unknown; "Quebec" refers to region of western Quebec serviced by CHEO; UK: United Kingdom; US: United States; CNS: central nervous system; LN: lymph node

**Table 3:** Characteristics of patients who did not meet the case definition

Case	Presentation	Investigations/course
1	Lung abscess	TST and TB cultures negative Improved on antibacterial therapy
2	Inpatient from Baffin Island TST 10 mm	No consistent symptoms CXR not suggestive, TB cultures negative
3	Abdominal pain TST 15 mm	Stool positive for *Ascaris Lumbricoides* Patient improved on anthelmintic therapy LTBI treated
4	Child from Baffin Island with respiratory symptoms Positive TST on history	No consistent symptoms, TST negative, CXR not suggestive, TB cultures negative
5	SVCO syndrome TST 20 mm	Mediastinal germ cell tumor diagnosed Cultures and pathology negative for TB LTBI treated
6	Abscess at BCG injection site	BCG abscess diagnosed
7	Acute bacterial pneumonia	TST and TB cultures negative Improved on antibacterial therapy
8	TB contact on history TST 6 mm	CXR: right hilar LAD No consistent symptoms, TB cultures negative (3 GWs), immunocompetent

LTBI: latent tuberculous infection; SVCO: superior vena cava obstruction syndrome; GW: gastric washing

During the first five-year period (April, 1998 - March, 2003), one (10%) of the 10 TB cases was from Baffin Island. This proportion rose to 60% during the second five-year period (April, 2003 - March, 2008). Overall, 7 (35%) of total inpatients were from Baffin Island. Nine (45%) were born in other countries. Seven children (35% of total patients) were from African nations, 5 of which were born in Somalia. All 7 African-born children were living in Ottawa at the time of diagnosis. Four of the Canadian-born children were residing in Quebec. The parents of these children were born in Canada, Haiti, Vietnam, and an unspecified African country.

BCG status was unknown in the majority. Eight (40%) were considered to have a history of BCG vaccination. A history of vaccination was obtained via immunization records in 3, the presence of a BCG scar in 2, and via history-taking in 3 children. Six (75%) of the 8 BCG recipients were from Baffin Island. Both children from Baffin Island who developed CNS TB had a history of BCG vaccination.

HIV status was known in 8 (40%) of patients. One of these 8 patients was HIV positive. This child developed abdominal TB and underwent excision of a tuberculous brain abscess. Her TB was treated and she subsequently did well on antiretroviral medications.

Contact with an infectious TB case was confirmed in 8 (40%) of cases. Contact was confirmed in 6 (85%) of 7 children from Baffin Island, and 2 (15%) of children from elsewhere.

No symptom or sign consistent with TB was present in more than 50% of patients. Fever was the most common complaint on history (50%), and the second most common objective finding (40%). Forty percent had a history of cough. The most common reported symptoms were constitutional, while the most common physical findings were abnormalities on chest examination, such as decreased air entry (45%) and rales (20%). Superficial lymphadenopathy was present in 3 children (15%), while erythema nodosum was found in one.

Fifteen children (75%) had low hemoglobin levels at presentation. Nine of these had a low MCV. Thus, 9 patients (45%) in the study met criteria for microcytic anemia, while 6 (30%) had a normocytic anemia.

A TST was done in 13 (65%) of children during their diagnostic evaluation. All of these children were found to be strongly positive, with reactions ranging from 14 to 40 mm. All patients had a chest radiograph done. Seventeen (85%) had at least one abnormality consistent with TB, including 3 of 6 children with extrathoracic disease sites. Hilar adenopathy was the most common finding, present in 9 (45%) of patients. Five children (25%) had a pleural effusion. Cavitation was seen on 2 films, and a miliary pattern on one.

*M. tuberculosis *was grown in culture from 20 specimens, taken from 15 patients (75%) who were culture positive from at least one site (Table 4). Cultures were positive from more than one site in 3 patients. Five isolates in total were resistant to antitubercular medications. Three isolates with single-agent resistance to isoniazid (INH) were grown from one patient. Two other patients had a resistant isolate, one to INH and another to streptomycin. There was no multi-drug resistance. All 3 children with drug-resistant TB were born in Africa.

**Table 4:** Positive microbiological testing

Specimen	Culture positive	Smear positive	PCR positive
Gastric aspirate	5	0	0
Expectorated sputum	3	3	3
Lymph node aspirate	3	3	2
Bronchial washings	3	0	0
Tracheal aspirate	1	0	0
Lumbar CSF	1	1	0
Brain biopsy	0	1	0
Pleural fluid	1	0	0
Ascitic fluid	1	0	0
Abscess aspirate	1	0	0
Stool	1	0	0

Total	20	8	5

Three children had sputum smear positive TB. Five specimens were positive on AMTD^®^, including 3 expectorated sputum samples and 2 lymph node aspirates. All specimens that were positive on AMTD^® ^were also both smear and culture positive.

Endothoracic disease occurred in 16 patients (80%), including 9 with primary pulmonary TB, 3 with "adult-type" disease, 3 with pleural TB, and one with miliary disease (Table 2). Extrathoracic disease was present in 6 children (30%). Disease sites included CNS disease (3), the abdomen (2), the spine (1), and superficial lymph node disease (1). Three children had disease in 2 separate sites.

There was a wide range of TB-related complications and surgical interventions in this series, despite the low number of cases. One child from Baffin Island developed corneal scarring secondary to phlyctenular conjunctivitis. Complications related to endothoracic lymph node compression included bronchial compression and esophageal ulceration. There were no cases of upper airway compromise. Two patients with pulmonary disease developed bronchiectasis. Hydrocephalus occurred in two patients with CNS disease; one developed the syndrome of inappropriate antidiuretic hormone secretion (SIADH), underwent ventriculostomy and later died. The other child developed a dystonic hemiparesis requiring physiotherapy and long-term neurology follow-up. A third child with CNS TB underwent excision of a brain abscess. The patient with spinal TB developed a psoas abscess and mild scoliosis. The most common surgical intervention was tube thoracostomy, performed in 4 children (20%).

## Discussion

Two main risk groups were identified in our study, namely children from Baffin Island and children of African origin. Together, these two groups contributed 70% of CHEO's TB inpatients between 1998 and 2008. We estimate that children aged 0-17 years in Baffin Island contribute roughly 1% of the CHEO catchment population. Meanwhile, 35% of TB cases between 1998 and 2008 came from this geographic area. The proportion of TB patients from Baffin Island rose from 10% during the first five-year period of the study to 60% during the second five-year period. Two of 3 children with CNS disease came from Baffin Island, along with the only death in the case series. Hospitalization data cannot be used to calculate incidence rates, but these data show clearly that Baffin Island makes a disproportionate contribution to our TB inpatient population.

African-born children may be exposed to TB in their country of origin or in Canada. Regardless of where the transmission occurs, it is the downstream effect of a TB/HIV pandemic in resource-poor nations. Conversely, children in Baffin Island are exposed as a result of disease and transmission occurring within Canada. The ongoing TB problem in northern Canada can be explained in part by history. Following contact with Europeans, TB rates in the Inuit population were among the highest ever reported [11]. The death rate in the Northwest Territories was 718 per 100, 000 in 1950. What followed were intense public health and medical interventions, which led to one of the most rapid rates of decline in TB incidence ever reported, approaching 20% per year in the 1970s [12]. Incidence in the Northwest Territories dropped to a low of 16 per 100, 000 in 1985, with a total of 9 reported cases overall [13].

There has been a resurgence of TB in northern Canada. Between 2004 and 2008, rates in Nunavut ranged between 99 and 184 per 100, 000, consistently more than 20 times the overall Canadian rate [14]. In 2007, the TB rate among children aged 0-14 years in Nunavut was 49 per 100, 000, 20 times higher than the overall Canadian rate for the same age group [15]. There is a large pool of latent tuberculous infection among the Inuit people, which persists among survivors of past epidemics. This case series provides evidence that new infections continue to occur in these communities, as pediatric TB is a good indicator of ongoing transmission. Furthermore, childhood cases from Baffin Island tend to be younger, reflecting the intensity of this transmission. In 2007, the TB rate among children aged 0-4 years in Nunavut was 112 per 100, 000, more than 40 times higher than the overall Canadian rate for the same age group [15].

All children living in Ottawa at the time of diagnosis were born in Africa. This reflects immigration patterns during the 1990s, particularly from war-torn nations like Somalia and Rwanda. The only child in our series with HIV co-infection was born in Africa, which is not unexpected given the HIV pandemic and its impact on TB in that part of the world [16]. The majority of TB cases reported in Canada are now foreign-born [15]. Due in part to their massive populations, India and China contribute the greatest numbers to the global TB burden. However, TB incidence rates are much higher in many African countries [17]. This is reflected in our national statistics: Asian-born communities contribute the greatest number of cases, whereas incidence rates are higher among African-born immigrants [15]. For the clinician, this translates into a higher individual risk among African-born children who live in Canada, when compared to children from other countries.

The secondary objective of this study was to describe the clinical manifestations, diagnostic methods, and clinical course of patients. Even with limited numbers, our data show that TB can present in many different ways, potentially leading to many different complications. Sensitivities of all symptoms and signs were low, confirming the need for good contact history, skin testing, radiology, and efforts to recover the organism. The latter was achieved in 75% of cases, which is quite high, and likely a result of relatively advanced illness (and presumably higher bacillary burden) and/or access to diagnostic services duration hospitalization. Both children from Baffin Island with CNS TB had a history of BCG vaccination, calling into question the efficacy of current BCG strains, and confirming the incomplete protection offered by this vaccine [18]. Complications secondary to lymph node compression and phlyctenular conjunctivitis - generally considered unique to childhood TB - were observed. The latter is known to occur at a higher than expected frequency among Inuit people, potentially leading to corneal scarring and visual loss [19]. Children presented with sputum smear positive "adult-type" disease. Cavitary disease with extensive transmission has been reported in a child as young as nine years old [20], and is not uncommon in the 10-14 year age group [21]. Anemia occurred in 75% of children. The role of iron supplementation is unclear in these patients, since *M. tuberculosis *requires iron for growth, and the anemia may simply resolve with treatment if it is secondary to TB. These observations open the door to a variety of research questions, although further discussion of their implications is beyond the scope of our paper.

The main limitations of the study were its low numbers, and all of the inherent limitations of retrospective chart review. Despite our small sample size, we believe these are important findings. TB has been recognized as an ongoing problem in northern Canada; this study provides further evidence in support of that fact, with a focus on affected children. This is an important observation in terms of being both a preventable disease occurring among children, and an indicator of ongoing TB transmission in northern Canada.

Another limitation in the study was its case definition. There is no gold standard for TB diagnosis in a live person, so studies of TB disease must adopt or formulate a case definition. We reviewed criteria used for diagnosis in both developed and developing countries, and chose fairly rigorous criteria. The down side of this approach is possible under-diagnosis, and we recognize that our case definition could lead to missed diagnoses and underestimation of TB burden in resource-poor settings. One of the children in Table 3 had supposed (unconfirmed) contact, CXR findings, and a 6 mm TST reaction. Due to lack of symptoms or a reason for anergy, we excluded this case from the series. The use of culture positivity alone as a criterion for diagnosis is also debated [22], although all of our culture-confirmed cases also had symptoms and other evidence of TB. The strength of our approach is that we are confident all of the children included in the study had TB. We believe such a case definition is appropriate in a setting of high resource availability.

## Conclusions

The results of this study suggest that transmission and disease are higher than expected among children from Baffin Island and among children of African origin. Health care providers working with these two communities should maintain a high index of suspicion for TB. Since Baffin Island is within Canada, the TB problem in this population warrants increased attention and public health measures to prevent transmission to children. Indeed, the cycle of transmission and disease in this population may represent the last reservoir of indigenous TB within our borders. The problem in Africa is associated with poverty and an HIV pandemic occurring outside Canada. While we must take measures to prevent TB among African-born children within our borders, long-term prevention will require our assistance internationally as well.

## Competing interests

The authors declare that they have no competing interests.

## Authors' contributions

Both authors contributed towards the conception and design of the study. The medical record abstraction form was developed by MC. Chart review, data entry and statistical analyses were carried out by MC. The article was drafted by MC and CH. Both also read and approved the final manuscript.

## Pre-publication history

The pre-publication history for this paper can be accessed here:

http://www.biomedcentral.com/1471-2431/10/102/prepub
